# Disease characteristics, effectiveness, and safety of vestronidase alfa for the treatment of patients with mucopolysaccharidosis VII in a novel, longitudinal, multicenter disease monitoring program

**DOI:** 10.1186/s13023-024-03176-z

**Published:** 2024-05-07

**Authors:** Roberto Giugliani, Antonio Gonzalez-Meneses, Maurizio Scarpa, Barbara Burton, Raymond Wang, Esmeralda Martins, Esmeralda Oussoren, Julia B. Hennermann, Brigitte Chabrol, Christina L. Grant, Angela Sun, Consuelo Durand, Joel Hetzer, Betsy Malkus, Deborah Marsden, J. Lawrence Merritt II

**Affiliations:** 1 Dep Genetics UFRGS, Casa dos Raros, INAGEMP, Med Genet Serv HCPA, and DASA Genomics, Porto Alegre, Brazil; 2 Hospital Universitario Virgen del Rocio, Seville, Spain; 3 Regional Coordinator Centre for Rare Diseases, University Hospital of Udine, Udine, Italy; 4 Feinberg School of Medicine, Northwestern University, Chicago, IL USA; 5 University of California Irvine School of Medicine, Children’s Health of Orange County, Orange, CA USA; 6 Centro Hospitalar Universitário de Santo António, Porto, Portugal; 7 Erasmus University Medical Center Rotterdam, Rotterdam, The Netherlands; 8 University Medical Center Mainz, Villa Metabolica, Mainz, Germany; 9 Centre Hospitalier Universitaire La Timone, Marseille, France; 10 Children’s National Hospital, Washington, District of Columbia, USA; 11 Seattle Children’s Hospital, Seattle, WA USA; 12 Laboratorio Chamoles, Buenos Aires, Argentina; 13 Ultragenyx Pharmaceutical Inc, Novato, CA USA

**Keywords:** Lysosomal storage disease, Non-immune hydrops fetalis, β-glucuronidase deficiency, Mucopolysaccharidosis VII, MPS VII

## Abstract

**Background:**

Mucopolysaccharidosis VII (MPS VII) is an ultra-rare, autosomal recessive, debilitating, progressive lysosomal storage disease caused by reduced activity of β-glucuronidase (GUS) enzyme. Vestronidase alfa (recombinant human GUS) intravenous enzyme replacement therapy is an approved treatment for patients with MPS VII.

**Methods:**

This disease monitoring program (DMP) is an ongoing, multicenter observational study collecting standardized real-world data from patients with MPS VII (*N* ≈ 50 planned) treated with vestronidase alfa or any other management approach. Data are monitored and recorded in compliance with Good Clinical Practice guidelines and planned interim analyses of captured data are performed annually. Here we summarize the safety and efficacy outcomes as of 17 November 2022.

**Results:**

As of the data cutoff date, 35 patients were enrolled: 28 in the Treated Group and seven in the Untreated Group. Mean (SD) age at MPS VII diagnosis was 4.5 (4.0) years (range, 0.0 to 12.4 years), and mean (SD) age at DMP enrollment was 13.9 (11.1) years (range, 1.5 to 50.2 years). Ten patients (29%) had a history of nonimmune hydrops fetalis. In the 23 patients who initiated treatment prior to DMP enrollment, substantial changes in mean excretion from initial baseline to DMP enrollment were observed for the three urinary glycosaminoglycans (uGAGs): dermatan sulfate (DS), -84%; chondroitin sulfate (CS), -55%; heparan sulfate (HS), -42%. Also in this group, mean reduction from initial baseline to months 6, 12, and 24 were maintained for uGAG DS (-84%, -87%, -89%, respectively), CS (-70%, -71%, -76%, respectively), and HS (+ 3%, -32%, and − 41%, respectively). All adverse events (AEs) were consistent with the known vestronidase alfa safety profile. No patients discontinued vestronidase alfa. One patient died.

**Conclusions:**

To date, the DMP has collected invaluable MPS VII disease characteristic data. The benefit-risk profile of vestronidase alfa remains unchanged and favorable for its use in the treatment of pediatric and adult patients with MPS VII. Reductions in DS and CS uGAG demonstrate effectiveness of vestronidase alfa to Month 24. Enrollment is ongoing.

**Supplementary Information:**

The online version contains supplementary material available at 10.1186/s13023-024-03176-z.

## Introduction

Mucopolysaccharidosis VII (MPS VII), or Sly syndrome, is a chronically debilitating, life-threatening lysosomal storage disorder exhibiting autosomal recessive inheritance [1, 2]. MPS VII is an ultra-rare disease; it is estimated there are 200 to 300 patients living with MPS VII worldwide [3]. MPS VII is caused by reduced activity of β-glucuronidase (GUS), which is encoded by the *GUSB* gene and required for degradation of the glycosaminoglycans (GAGs) dermatan sulfate (DS), chondroitin-6-sulfate (CS), and heparan sulfate (HS). GUS deficiency leads to lysosomal accumulation of GAGs in multiple tissues and organs throughout the body, resulting in numerous clinical symptoms as a result of tissue damage and organ dysfunction [4, 5]. 

Features of MPS VII can present in the prenatal or postnatal period. Patients with MPS VII may present with non-immune hydrops fetalis (NIHF) leading to a number of physiological complications [6]. Most patients with MPS VII have cognitive impairment manifesting as developmental delay, language delay, and intellectual impairment [4]. Other common characteristics of MPS VII include abnormal coarsened facies, cardiac disease, pulmonary disease, hepatomegaly, splenomegaly, severe joint and bone abnormalities, short stature, and corneal clouding. The rate of MPS VII disease progression is variable and likely multifactorial; many patients with MPS VII die from the disease before the second or third decade of life due to comorbid medical issues [4]. 

Vestronidase alfa is a formulation of recombinant human β-glucuronidase intended as enzyme replacement therapy for patients with MPS VII. The efficacy and safety of vestronidase alfa were consistently shown across all phases of the MPS VII clinical development program (Phase 1/2 trial ClinicalTrials.gov Identifier: NCT01856218, Phase 2 trial ClinicalTrials.gov Identifier: NCT02418455, Phase 3 trial ClinicalTrials.gov Identifier: NCT02432144, and Phase 3 trial ClinicalTrials.gov Identifier: NCT02230566) [7, 8]. In the phase 3 trial with a blind-start study design in patients with MPS VII, 10 of 12 participants showed meaningful improvement in at least one domain of a Multi-domain Responder Index (MDRI) after 24-weeks of treatment with vestronidase alfa [7]. Based on the positive outcomes observed in these trials, vestronidase alfa was approved by health authorities in the United States, Europe, Japan, and Latin America for the treatment of pediatric and adult patients with MPS VII.

Given the rarity of this disease and the somewhat recent availability of an approved therapy, there is a critical need to increase disease awareness and impart the importance of early diagnosis in the medical community. It is also critical that healthcare providers understand the clinical heterogeneity of MPS VII to better characterize the clinical presentation and progression, both in patients treated and untreated with vestronidase alfa.

The MPS VII Disease Monitoring Program (DMP) is a unique prospective longitudinal study to characterize MPS VII disease presentation and progression over time in all patients, both treated and untreated with vestronidase alfa therapy (ClinicalTrials.gov Identifier: NCT03604835). The DMP is assessing the long-term effectiveness and long-term safety of vestronidase alfa in patients with MPS VII and is also investigating longitudinal changes in biomarkers, clinical assessments, and patient/caregiver-reported outcome measures. The comprehensive study design of the DMP will provide greater insight over a traditional registry into the effects of this ultra-rare disease on participants. Here, we present the initial demographics, disposition, immunogenicity, hypersensitivity, adverse events, and efficacy outcomes from the DMP through November 2022.

## Methods

### DMP study design

The DMP is a global, prospective, multicenter, longitudinal study to characterize MPS VII disease presentation and progression. Long-term effectiveness and safety of vestronidase alfa, including hypersensitivity reactions and immunogenicity, will be monitored. Longitudinal changes in biomarkers, clinical assessments, patient/caregiver-reported outcome measures, and other possible predictors of MPS VII disease progression and mortality will also be assessed. The protocol was approved by the institutional review boards at respective sites.

### Enrollment, site selection, and training

Target enrollment is approximately 50 participants with MPS VII globally across 15 sites in the United States, Brazil, The Netherlands, France, Germany, Spain, Portugal, Argentina, and Türkiye; remote enrollment is also available. All program sites are academic centers with experience assessing and managing patients with MPS VII. Sites were trained on assessment measures to facilitate standardization and decrease variability; sites are compensated for their efforts, as applicable, based on local laws and regulations. Vestronidase alfa is not provided as part of the study, though participants may receive vestronidase alfa either through authorized commercial use or available early access programs.

### Data handling, record keeping, and retention

Data are captured using a validated electronic data capture system and are source verified for completeness and accuracy based on standard Good Clinical Practice (GCP) monitoring methods. Monitoring and auditing procedures are being implemented to ensure compliance with International Conference on Harmonisation GCP guidelines. Interim analyses of data captured in the DMP are planned annually. An advisory committee was formed in 2018 to provide guidance on overall program design and conduct, as well as data dissemination and publications.

### Eligibility criteria

The MPS VII DMP is designed to be inclusive, with no restrictions on treatment status. Participants must have a confirmed diagnosis of MPS VII based on enzymatic laboratory diagnosis confirmed by molecular testing. Prior to any research-related procedures, participants must provide written informed consent or, in the case of participants < 18 years of age (or 16 years, depending on the region) or participants > 18 years of age with cognitive deficiencies, provide written assent (if required), with written informed consent provided by a legally authorized representative after the nature of the DMP has been explained. Eligible participants also had to be willing to comply with the DMP visit schedule.

Exclusion criteria include concurrent enrollment in an Ultragenyx-sponsored clinical trial and participation in any other biopharmaceutical company-sponsored interventional clinical trial, unless approved by Ultragenyx.

### Study visits and assessments

Enrolled participants have annual in-clinic visits at study sites. Biannual visits are planned in the first two years for participants < 5 years and in the first year for participants ≥ 5 years. Phone calls to both the treating physician (if different than the DMP investigator) and the participant/caregiver assess potentially unreported safety information.

Age-appropriate assessments (Table 1) are collected at specified intervals, depending on participant age at the time of the visit. Families may receive travel assistance (with certain restrictions) to facilitate participant retention. Individual participant data are also shared at the annual visits with respective DMP investigators and participants and caregivers to allow physicians, participants, and caregivers to see how they are contributing to the overall DMP and their individual change over time.

Key assessments include functional assessments of cognition, mobility, skeletal disease, and pulmonary and cardiac function, and clinical assessment of uGAGs. Participant and caregiver-reported outcomes, including assessments of fatigue, activities of daily living, work productivity, and general health-related quality of life, are recorded at each visit.

All participants are encouraged to complete all data collection that is safe and reasonable, considering their specific condition and situation. The protocol was amended in 2021 to allow for remote enrollment, remote visits, or both, to help address COVID-19-related protocol deviations.

Efficacy and long-term safety data are captured for participants receiving vestronidase alfa therapy. In addition, the immunogenicity profile of vestronidase alfa is collected at each scheduled visit. The study is not randomized. With the small sample size and the heterogeneity among participants with MPS VII, no formal statistical testing is planned.

**Table 1:** Key Assessments

Assessment Type	Measures
General	Demographics, Physical Examination, Family History, Medical History, Diagnostic History, Clinical Laboratory Tests (including uGAG and anti-drug antibody testing), growth (height, weight, head circumference)
Adaptive Behavior and Cognitive Assessment	Vineland Adaptive Behavior Scales 3rd edition and Cognitive Assessments
Motor Function	Gross Motor Milestone Checklist (< 5 years of age)
Mobility and Endurance	Six-minute walk test (6MWT; ≥5 years of age)
Patient/Caregiver Reported Outcomes	Pediatric Quality of Life Inventory™ (PedsQL)-Multidimensional Fatigue Scale (≥ 2 years of age), EuroQol Five Dimensions Questionnaire Five Levels (EQ-5D-5L), MPS Health Assessment Questionnaire (≥ 5 years of age), Work Productivity and Activity Impairment (WPAI) Questionnaire, and Caregiver Self-report
Pulmonary Function	Spirometry (≥ 5 years of age), Cough peak flow (≥ 4 years of age)
Cardiac Function	Electrocardiogram, 2D-echocardiogram
Visual Acuity	Snellen Eye Chart (≥ 5 years of age)
Hearing Impairment	Audiometry

## Results

### Participant disposition and demographics

Informed consent for the first participant was provided on 29 January 2018. As of the data cutoff for this analysis (17 November 2022), 35 participants were enrolled as follows: 11 in the United States, nine in Brazil, five in Argentina, three in France, three in Portugal, two in the Netherlands, and two in Spain. Seven participants have not been treated with vestronidase alfa and are enrolled in the Untreated Group. Among the 28 participants in the Treated Group, 23 initiated vestronidase alfa treatment prior to enrollment in the DMP (Treated Prior to DMP Enrollment Group) and the remaining five initiated treatment after enrollment (Treated After DMP Enrollment Group).

Thirty-four enrolled participants continue in the DMP as of the data cutoff; one participant in the Treated Prior to DMP Enrollment Group died due to a recreational activity accident. Mean (SD) duration of enrollment to last follow-up date was 1.5 (1.2) years, and the total participant-years of enrollment in the DMP was 52.0. Of the 23 participants in the Treated Prior to DMP Enrollment Group, 16 of 19 completed a Month 6 Visit (meaning 16 out of a potential 19 patients reached the 6-month mark and had a Month 6 visit), 16 of 18 completed a Month 12 Visit, eight of eight completed a Month 24 Visit, four of five completed a Month 36 Visit, and two of two completed a Month 48 visit. Of the five participants in the Treated After DMP Enrollment Group, two had a DMP visit after initiating vestronidase alfa, and three had a baseline visit but no post-baseline visits. Of the seven participants in the Untreated Group, six of seven completed a Month 6 Visit, two of two completed a Month 12 Visit, one of one completed a Month 24 Visit, and one of one completed a Month 36 Visit. The mean (SD) age of participants at enrollment into the DMP was 13.9 (11.1) years, with a range of 1.5 to 50.2 years. Broad heterogeneity was observed in MPS VII pathogenic variants across participants enrolled in the DMP (Table 2).

**Table 2:** Participant Demographics

Parameter	Total (*N* = 35)
**Age at enrollment, mean (SD)** **[min, max], years**	13.9 (11.1) [1.5, 50.2]
< 12 years, n (%)	18 (51)
≥ 12 years, n (%)	17 (49)
**Sex, n (%)**	
Male	19 (54)
Female	16 (46)
**Race, n (%)**	
White	16 (46)
American Indian/Alaska Native	4 (11)
Black/African American	5 (14)
Unknown or not reported	6 (17)
Asian	3 (9)
Multiple	1 (3)
Native Hawaiian / Other Pacific Islander	0 (0)
**Ethnicity, n (%)**	
Hispanic or Latino	14 (40)
Not Hispanic or Latino	17 (49)
Unknown or not reported	4 (11)
MPS VII pathogenic variants*, n (%)	
c.526 C > T p.(Leu176Phe) and c.526 C > T p.(Leu176Phe)	7 (20)
p.(Leu322Phe) and p.(His127Arg)	2 (6)
c.526 C > T p.(Leu176Phe) and c.875T > C p.(Leu292Pro)	1 (3)
c.526 C > T p.(Leu176Phe) and unknown	1 (3)
p.(Arg122Ser) and p.(Asn502Lys)	3 (9)
c.1222 C > T and c.1244 C > T	3 (9)
c.161 A > G and c.1120 C > T	2 (6)
c.295 > A p.(Val99Met) and unknown	2 (6)
c.88 C > T p.(Pro30Ser) and c.290G > C p.(Gly97Ala)	1 (3)
c.863G > T p.(Trp288Lys) and c.1454 C > T p.(Ser485Phe)	1 (3)
c.499 C > G and c.1244 + 3G > C	1 (3)
c.200 C > T and c.1325 C > T	1 (3)
c.431G > C and c.431G > C	1 (3)
c.1412 A > C and c.1412 A > C	1 (3)
c.1486G > T and c.1760G > C	1 (3)
c.1818G > C and c.1818G > C	1 (3)
p.(Thr177Ile) and p.(Thr177Ile)	1 (3)
No mutation reported in DMP	5 (14)

*Pathogenic variant data as of 17NOV2023. Information may be incomplete and data collection are ongoing.

### Coronavirus disease 2019 (COVID-19) impact

Travel restrictions related to the global COVID-19 pandemic resulted in unavoidable protocol deviations. A total of 29 COVID-19-related protocol deviations occurred in 13 participants: three visits occurred out of the scheduled window, 21 visits were not performed, and five assessments for three participants were not performed. One dose of vestronidase alfa was missed due to family members of a participant testing positive for COVID-19.

### Medical and surgical history

The most frequently reported conditions noted in medical history assessments were in the Medical Dictionary for Regulatory Activities System Organ Classes of surgical and medical procedures (15/35, 43%), infections and infestations (14/35, 40%), and congenital familial and genetic disorders (11/35, 31%). The most frequent Preferred Terms of conditions noted in general medical history assessments were rhinitis (6/35, 17%), central venous catheterization (5/35, 14%), and hepatomegaly (5/35, 14%). Other than the previously noted central venous catheterization, additional surgical and medical procedures included ear tube insertion (3/35, 9%), spinal laminectomy (3/35, 9%), inguinal hernia repair (3/35, 9%), gastrostomy (2/35, 6%), spinal operation (2/35, 6%), tonsillectomy (2/35, 6%), transfusion (2/35, 6%), and umbilical hernia repair (2/35, 6%).

Most patients with MPS VII reported in the literature have cognitive disabilities manifesting as developmental delay, language delay, and intellectual impairment, which can affect their ability to complete certain clinical assessments [4]. In the DMP, intellectual disability was reported for 24 participants (20 in the Treated Group and four in the Untreated Group) and cognitive development data were reported as unknown or not applicable for 11 participants. Of the 24 participants with intellectual impairment, severity was reported as profound for five participants, severe for six, moderate for three, and mild for six. The four remaining participants had documented intellectual disability with unspecified severity. For the 11 participants with unknown or not applicable cognitive development data, efforts to collect this information are ongoing and may be available as the DMP progresses.

Hydrops fetalis was noted for two participants; however, features suggestive of hydrops fetalis were noted for 16 participants, including symptoms reported at birth of anasarca, ascites, pericardial and pleural effusion, and abdominal distension. Most participants either had unknown birth history or had normal full-term delivery. Three participants were born pre-term at 34, 35, 37 weeks, and one participant reported to have been born preterm, but the week of birth was not specified.

### Vestronidase alfa exposure

Vestronidase alfa was administered at the standard dose of 4 mg/kg every two weeks (Q2W) to most participants. Mean (SD) age at initiation of vestronidase alfa for the participants treated prior to DMP enrollment was 8.1 (6.0) years (range: 1.0 to 22.8 years), and mean (SD) duration of treatment with vestronidase alfa up to the last follow-up was 5.1 (2.4) years (range: 0.1 to 8.4 years; Table 3).

**Table 3:** Vestronidase Alfa Exposure in Participants Treated Prior to DMP Enrollment

	Total Treated (*N* = 23)
Age at Initiation of Vestronidase Alfa Treatment n mean (SD), years range, years	21 8.1 (6.0) 1–22.8
Duration Since Initiation of Vestronidase Alfa to the Last Follow-up n mean (SD), years range, years	21 5.1 (2.4) 0.1–8.4
Duration Since the First Vestronidase Alfa During DMP to the Last Follow-up n mean (SD), years range, years	23 1.6 (1.3) 0.0–4.5

n: number of participants with available data; SD: standard deviation

### Safety and hypersensitivity reactions

Serious hypersensitivity reaction is an important potential risk for vestronidase alfa. As of the cutoff date, no serious hypersensitivity reactions were identified, and no participants discontinued the study or vestronidase alfa due to adverse events. One participant died from a recreational activity accidental death that was considered unrelated to vestronidase alfa by the investigator and the sponsor. Additionally, no association between anti-drug-antibodies (ADA) or neutralizing antibodies (NAb) and serious hypersensitivity reaction risk could be determined. There were no observed associations between hypersensitivity reactions and ADA or NAb against vestronidase alfa, *GUSB* genotype, or intrinsic GUS enzymatic activity.

### Incidence of ADA and NAb against vestronidase alfa

As of the data cutoff date for this analysis, 92 ADA samples from 26 participants were collected and analyzed, including 28 samples collected prior to enrollment into the DMP. No samples were collected from eight participants (ADA sample collection is either not in the country-specific protocol or not standard of care). A participant was classified as ADA-positive only if the participant showed at least a 4-fold increase from their baseline titer or if the participant showed a post-baseline titer of at least 1:40 or above when no antibody was detected at baseline.

Four participants tested positive at initial baseline, with titers ranging from 1:80 to 1:160. None of these four participants tested positive for ADA at any subsequent DMP Visits (including the DMP Enrollment Visit). Seven participants tested positive for ADA during the DMP, with titers ranging from 1:40 to 1:320. If a sample tested positive for ADA, then the sample was tested for NAbs against vestronidase alfa. Two positive ADA samples tested positive for NAbs; to date, there has been no loss of efficacy reported in these participants.

### Other adverse events

Fourteen serious adverse events (SAEs) were reported in nine participants. Three adverse events in two participants were considered related to vestronidase alfa: there was one SAE of intermittent hypotension, and two nonserious AEs of persistent hypotension (moderate, resolved) and low-grade fever (mild, resolved). All other SAEs, which included recurrent cervical spinal stenosis, corneal opacity, injury from a fall, pneumonia, chorioretinal scar, recreational activity accident, acute hypoxemic respiratory failure, clonus, and parainfluenza virus infection, were considered unrelated to vestronidase alfa. No participants experienced cervical/spinal cord compression during the DMP as of this data cutoff; however, patients with MPS VII should be carefully monitored on an ongoing basis for spinal stenosis and compression.

### Vestronidase alfa efficacy

**Fig. 1:**
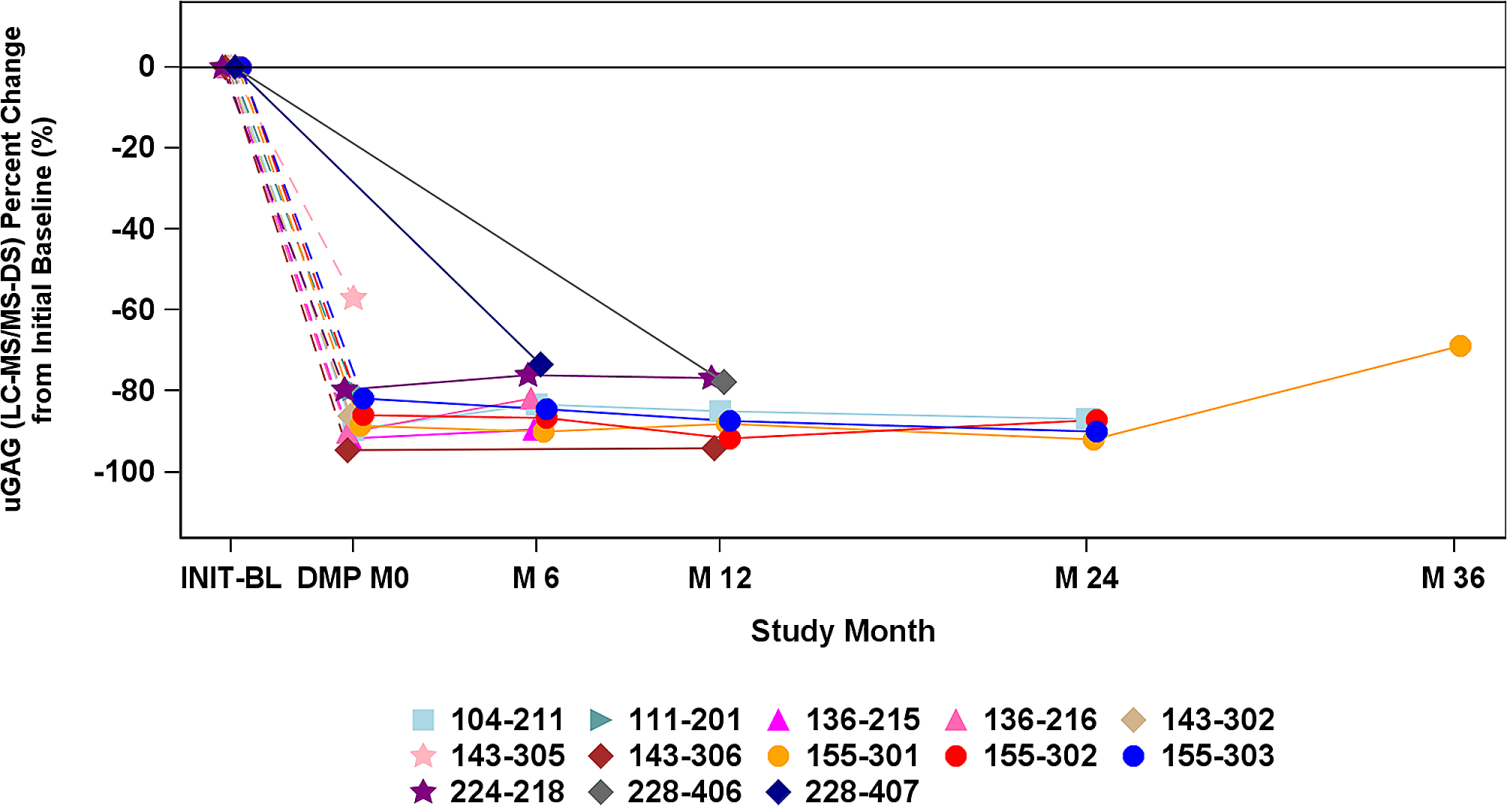
Dermatan Sulfate Excretion in Participants Treated With Vestronidase Alfa. INIT-BL: initial baseline. For the 11 participants with values at INIT-BL, the range from INIT-BL to DMP M0 was 947 to 2631 days

Excretion of uGAGs is a direct pathophysiological marker of the MPS disease process and serves as a key efficacy measure for vestronidase alfa treatment [9]. For participants in the Treated Prior to DMP Enrollment Group (*n* = 23), substantial changes in mean excretion from initial baseline to DMP Enrollment were observed for all three uGAGs: DS, -84%; CS, -55%; HS, -42%. Assessed relative to the upper limit of normal (ULN), mean excretion in the Treated Prior to DMP Enrollment Group decreased from initial baseline to DMP enrollment for DS from 23.26x to 4.23x ULN and for CS from 14.97x to 10.47x ULN; while the percent change from baseline decreased for HS, the changes were within the normal range, so assessments relative to the ULN are not clinically meaningful. Also in the Treated Prior to DMP Enrollment Group, mean reduction from initial baseline to Months 6, 12, 24, and 36 were maintained for DS (-84%, -87%, -89%, and − 69%, respectively; Fig. 1), CS (-70%, -71%, -76%, and − 72%, respectively), and HS (+ 3%, -32%, -41%, and − 22%, respectively).

In the Treated After DMP Enrollment Group (*n* = 5), these participants showed elevations of DS and CS (approximately 11x and 18x, respectively). Only two participants had a DMP visit occur after initiating vestronidase alfa: one participant had visits at Months 6 and 24 while receiving vestronidase alfa, and another participant was receiving vestronidase alfa at Month 12 but was not receiving treatment at Months 24 and 36. More recent data have not been reported for these participants as of the data cutoff for this analysis, and compared with untreated participants, baseline values were comparable. Response to vestronidase alfa treatment will continue to be closely followed for participants treated after DMP enrollment.

Only three participants had data reported from the Untreated Group. At DMP enrollment, mean DS, CS, and HS values were approximately 9x, 21x, and 1x ULN, respectively. One participant had Month 12 DS, CS, and HS values of 9x, 19x, and 1x ULN, respectively, findings consistent with the expected fluctuation of uGAG levels in an untreated patient with MPS VII.

### Height

All seven participants in the Untreated Group had data at DMP enrollment for recumbent length/standing height. In the Treated Prior to the DMP Enrollment Group (*N* = 23), recumbent length/standing height values were available at DMP enrollment and at initial baseline for 18 participants; in this same group, Z scores were available at DMP enrollment and at initial baseline for 15 and 17 participants, respectively. All five participants in the Treated After DMP Enrollment Group had recumbent length/standing height values and Z scores.

Mean (SD) recumbent length/standing height Z scores at DMP enrollment for the Treated Prior to DMP Enrollment and the Treated After DMP Enrollment Groups were − 2.40 (2.15) and − 2.61 (1.09), respectively. Initial baseline and post-DMP enrollment values were similar to DMP enrollment values, with no trends evident from the limited data available as of the data cutoff for this report.

**Fig. 2:**
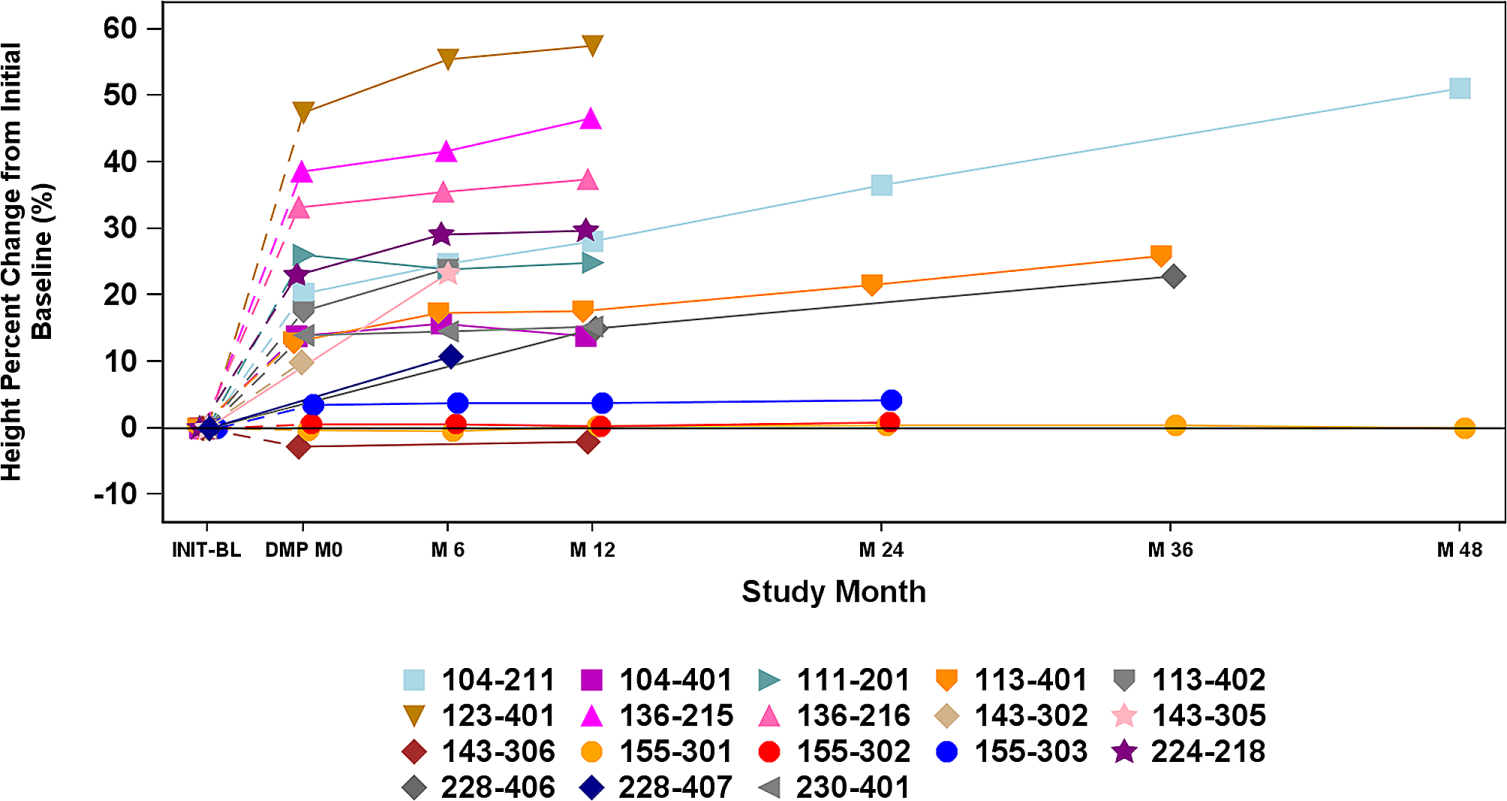
Percent Change From Initial Baseline in Height by Visit

Percent change from initial baseline in height by visit for participants treated with vestronidase alfa are shown in Fig. 2.

### Six-minute walk test

Data for the six-minute walk test (6MWT) were available for 21 participants as of the data cutoff for this analysis. The 6MWT was conducted at DMP enrollment for 11 participants in the Treated Prior to DMP Enrollment Group, one participant in the Treated After DMP Enrollment Group, and four participants in the Untreated Group. Eight participants had initial baseline assessments (seven participants in the Treated Prior to DMP Enrollment Group and one participant in the Treated After DMP Enrollment Group). Distance walked in the 6MWT at DMP enrollment for the treated group ranged from 139 to 453 m; mean (SD) percent predicted distance walked in the 6MWT normative matched for age, sex, and height was 52.2 (21.0) percent [10, 11]. 

In the Treated Prior to DMP Enrollment Group, 6MWT assessments were available at Months 6, 12, 24, 36, and 48 for nine, ten, five, two, and two participants, respectively. Participant assessments during the 6MWT showed no significant clinical change, other than one participant showing a decrease in pulse oximetry (SpO2) at baseline that was not observed in subsequent assessments.

### Pulmonary function

Pulmonary function data were collected at the DMP enrollment visit for 12 participants and post-DMP enrollment visits for eight participants. Thirteen of the 32 participants with data available were unable to follow instructions for the pulmonary function assessment, and one participant could not perform the assessments because this participant was dependent on a ventilator. Intellectual disability was reported for most participants, which may have impaired their ability to understand instructions for this assessment.

While the limited data available as of the data cutoff for this report prevents detailed interpretation, the eight participants with post-DMP enrollment visits remain within normal predicted ranges, with stable to slightly improved trends on the volume of air that can be forcibly exhaled in the first second after full inspiration (FEV1) and forced vital capacity (FVC). As an example, in one participant in the Treated Prior to DMP Enrollment Group, FEV1 was 3.29 at DMP enrollment versus a predicted FEV1 of 3.47; at Month 12, FEV1 increased to 3.64 versus a predicted FEV1 of 3.57. In another participant in the Treated Prior to DMP Enrollment Group, FVC was 0.86 at DMP enrollment versus a predicted FVC of 0.80; at Month 36, FVC increased to 1.28 versus a predicted FVC of 1.03. Six participants had post-baseline cough peak flow (CPF) assessments, and generally improved trends in CPF were observed with vestronidase alfa treatment. In a participant in the Treated Prior to DMP Enrollment Group, CPF was 2.45 at DMP enrollment; at Month 12, CPF increased to 3.06. The trends observed in FEV1, FVC, and CPF will continue to be closely monitored.

### Echocardiogram

Medical history collected for the vestronidase alfa treated group (*n* = 28) indicated that aortic, mitral, and tricuspid valve incompetence were present in seven (25.0%), four (14.3%), and two (7.1%) participants, respectively. Aortic valve thickening was reported in two (7.1%) participants, and mitral valve thickening was also reported in two (7.1%) participants.

Collection of echocardiogram data was highly variable through the study to date; not all data were collected due to regional differences in care. Left ventricular mass index (LVMI) data were available at DMP enrollment for 12 participants in the Treated Prior to DMP Enrollment Group, five participants in the Treated After DMP Enrollment Group, and two participants in the Untreated Group. Data for LVMI were also available at Month 12 for the Treated Prior to DMP Enrollment Group and the Untreated Group, and at Month 24 and Month 36 for the Treated Prior to DMP Enrollment Group. Although limited data available as of this report prevents a more detailed interpretation, some decreases in LVMI were reported in participants treated with vestronidase alfa; mean (SD) change from baseline at Month 24 in two participants in the Treated Prior to DMP Enrollment Group was − 22.8 (9.2). These trends will continue to be followed as more data become available.

## Discussion

The MPS VII DMP has started to provide a prospective, comprehensive, and standardized dataset for this ultra-rare and heterogeneous disease. The information available as of the data cutoff for this manuscript does not impact the known safety profile of vestronidase alfa, which includes identified risks of infusion site extravasation, diarrhea, rash, anaphylaxis, infusion site swelling, peripheral swelling, and pruritus. No new vestronidase alfa safety concerns or safety signals were identified since the initiation of the DMP. Based on all available efficacy and safety data for vestronidase alfa, the overall benefit-risk profile remains positive in the approved indication.

Similar to observations from the vestronidase alfa clinical program, substantial reductions (≥ 50%) in uGAGs DS and CS occurred in participants in the Treated After DMP Enrollment Group and were maintained for all participants in the Treated Prior to DMP Enrollment Group. Such findings demonstrate consistent and long-term efficacy with vestronidase alfa for up to nine years (including time in previous trials and time in the DMP) in this progressively debilitating disease. The efficacy data collected to date suggest no changes to the characterization of vestronidase alfa efficacy following additional exposure.

The MPS VII DMP includes patient and caregiver outcome assessments using the PedsQL-Multidimensional Fatigue Scale, the EQ-5D-5L, the MPS Health Assessment Questionnaire for participants ≥ 5 years of age, and the WPAI Questionnaire. Data from these assessments were only available from a limited number of participants as of the data cutoff date for this analysis; however, data collection for these parameters is ongoing and will provide insights into real-world outcomes for participants with MPS VII enrolled in the DMP as the study continues.

This DMP is also providing a substantial amount of MPS VII disease history data that may inform management strategies across a broad range of patients with MPS VII. The heterogeneous presentation of this condition and broad range of complication severities can be challenging for caregivers and clinicians. It is anticipated that as more data are collected over the long-term duration of the DMP, greater insight into the progression of disease and any impact of vestronidase alfa on slowing progression may become apparent.

Limitations of the MPS VII DMP include that patients with severe MPS VII may not survive to be enrolled or may be too ill to participate. As a result, the participants enrolled and contributing data to the DMP may be more mild cases of MPS VII compared with those unable to participate due to severe disease complications.

## Conclusion

The MPS VII DMP continues to enroll participants and collect data to characterize MPS VII disease presentation, clinical heterogeneity, and progression, as well as to assess any changes to the positive benefit-risk profile of vestronidase alfa. The MPS VII DMP will provide a greater understanding of MPS VII progression and response to enzyme replacement therapy as more participants are enrolled and evaluated over time. This novel approach may overcome issues encountered by previous patient registries and improve outcomes for patients with MPS VII.

### Electronic supplementary material

Below is the link to the electronic supplementary material.

Supplementary Material 1

## Data Availability

The dataset presented in this manuscript is available from the corresponding author (mailto: rgiugliani@hcpa.edu.br) or Ultragenyx Pharmaceutical Inc. (mailto: publications@ultragenyx.com) on reasonable request. Study subject identification numbers and other personally identifiable information will be redacted to protect participant privacy.

## Abbreviations

ADA: anti-drug-antibodies
COVID-19: Coronavirus Disease 2019
CPF: cough peak flow
CS: chondroitin-6-sulfate
DMP: Disease Monitoring Program
DS: dermatan sulfate
EQ-5D-5L: EuroQol Five Dimensions Questionnaire Five Levels
FEV1: volume of air that can be forcibly exhaled in the first second after full inspiration
FVC: forced vital capacity
GAGs: glycosaminoglycans
GCP: Good Clinical Practice
GUS: β-glucuronidase
HS: heparan sulfate
LVMI: left ventricular mass index
MDRI: Multi-domain Responder Index
MPS VII: Mucopolysaccharidosis VII
NAb: neutralizing antibodies
NIHF: non-immune hydrops fetalis
PedsQL: Pediatric Quality of Life Inventory
Q2W: every two weeks
SAEs: serious adverse events
SD: standard deviation
6MWT: Six-minute walk test
SpO2: pulse oximetry
WPAI: Work Productivity and Activity Impairment
ULN: upper limit of normal
