# Supplementary material for: Disease characteristics, effectiveness, and safety of vestronidase alfa for the treatment of patients with mucopolysaccharidosis VII in a novel, longitudinal, multicenter disease monitoring program
Source: Orphanet J Rare Dis. 2024 May 7;19:189. doi: 10.1186/s13023-024-03176-z (PMC11077874; doi:10.1186/s13023-024-03176-z)
Supplement: Supplementary file 1 — Supplementary Material 1 [file 13023_2024_3176_MOESM1_ESM.docx]

---------- Forwarded message ---------
De: **Orphanet Journal of Rare Diseases Editorial Office** <[em@editorialmanager.com](mailto:em@editorialmanager.com)>
Date: qua., 31 de jan. de 2024 às 05:56
Subject: Your submission to Orphanet Journal of Rare Diseases - OJRD-D-23-01006
To: Roberto Giugliani <[rgiugliani@hcpa.edu.br](mailto:rgiugliani@hcpa.edu.br)>

OJRD-D-23-01006
Disease characteristics, effectiveness, and safety of vestronidase alfa for the treatment of patients with mucopolysaccharidosis VII in a novel, longitudinal, multicenter Disease Monitoring Program
Roberto Giugliani; Antonio Gonzalez-Meneses; Maurizio Scarpa; Barbara Burton; Raymond Wang; Esmeralda Martins; Esmeralda Oussoren; Julia B. Hennermann; Brigette Chabrol; Christina L. Grant; Angela Sun; Consuelo Durand; Joel Hetzer; Betsy Malkus; Deborah Marsden; J. Lawrence Merritt
Orphanet Journal of Rare Diseases

Dear Prof. Giugliani,

Your manuscript 'Disease characteristics, effectiveness, and safety of vestronidase alfa for the treatment of patients with mucopolysaccharidosis VII in a novel, longitudinal, multicenter Disease Monitoring Program' (OJRD-D-23-01006) has been assessed by our reviewers. Although it is of interest, we are unable to consider it for publication in its current form. The reviewers have raised a number of points which we believe would improve the manuscript and may allow a revised version to be published in Orphanet Journal of Rare Diseases.

Their reports, together with any other comments, are below. Please also take a moment to check our website at [https://www.editorialmanager.com/ojrd/](https://protect-usb.mimecast.com/s/0ayYCoAWWZUrwA0qu1Nn2w?domain=editorialmanager.com/) for any additional comments that were saved as attachments.

If you are able to fully address these points, we would encourage you to submit a revised manuscript to Orphanet Journal of Rare Diseases. Once you have made the necessary corrections, please submit online at:

Your username is: RGiugliani-825

If you forgot your password, you can click the 'Send Login Details' link on the EM Login page at [https://www.editorialmanager.com/ojrd/](https://protect-usb.mimecast.com/s/0ayYCoAWWZUrwA0qu1Nn2w?domain=editorialmanager.com/).

Please include a point-by-point response within the 'Response to Reviewers' box in the submission system and highlight (with 'tracked changes'/coloured/underlines/highlighted text) all changes made when revising the manuscript. Please ensure you describe additional experiments that were carried out and include a detailed rebuttal of any criticisms or requested revisions that you disagreed with. Please also ensure that your revised manuscript conforms to the journal style, which can be found in the Submission Guidelines on the journal homepage.

The due date for submitting the revised version of your article is 01 Mar 2024.

Please note, if your manuscript is accepted you will not be able to make any changes to the authors, or order of authors, of your manuscript once the editor has accepted your manuscript for publication. If you wish to make any changes to authorship before you resubmit your revisions, please reply to this email and ask for a 'Request for change in authorship' form which should be completed by all authors (including those to be removed) and returned to this email address. Please ensure that any changes in authorship fulfil the criteria for authorship as outlined in BioMed Central's editorial policies ([http://www.biomedcentral.com/about/editorialpolicies#authorship](https://protect-usb.mimecast.com/s/cb20CypWWwSr4GRXuyqNa2?domain=biomedcentral.com)).

Once you have completed and returned the form, your request will be considered and you will be advised whether the requested changes will be allowed.
By resubmitting your manuscript you confirm that all author details on the revised version are correct, that all authors have agreed to authorship and order of authorship for this manuscript and that all authors have the appropriate permissions and rights to the reported data.

Please be aware that we may investigate, or ask your institute to investigate, any unauthorised attempts to change authorship or discrepancies in authorship between the submitted and revised versions of your manuscript.

I look forward to receiving your revised manuscript soon.

Best wishes,

Agnieszka Ługowska
Orphanet Journal of Rare Diseases
[https://ojrd.biomedcentral.com/](https://protect-usb.mimecast.com/s/9TINCqAWWQU82go0fQIrKz?domain=ojrd.biomedcentral.com/)


Reviewer reports:

Reviewer #1: This manuscript "Disease characteristics, effectiveness, and safety of vestronidase alfa for the treatment
of patients with mucopolysaccharidosis VII in a novel, longitudinal, multicenter Disease Monitoring Program"
describes  longitudinal (but intermediary) data over 48 months max, regarding an ultra-rare lysosomal disease, pooling untreated and ERT treated patients around the world.

Neverteless, this concerns safety data (comparable to those described before in trials) and longitudinal biological data focused on biomarqueurs Urinary GAGS, adding a follow-up on ERT > 8  years for x patients.
Clinical efficiency reported is a stabilisation trend or light improvement trend concerning respiratory and cardiac function for a few patients (without precision on ages of these patients) .
The title , and the discussion should give this precision  : Disease characteristics, BIOLOGICAL sustained effectiveness, and safety of vestronidase alfa for the treatment
of patients with mucopolysaccharidosis VII in a novel, longitudinal, multicenter Disease Monitoring Program


Some informations could be added :
- hearing/audiometry is announced as a key assessment in table 1 ,  and could impact language / cognitive evaluation

***Audiometry data are not available as of yet for this group of participants enrolled in the Disease Monitoring Program (DMP). Additional data will be collected as participants continue in the DMP.***

- cognitive data "unknown" or "not applicable" for 11  patients : what does "not applicable" mean:  patients have normal cognition ? or data not available = unknown.

***Cognitive development data are reported for 24 participants with this information available. The following sentence was added to this paragraph:***

For the 11 participants with unknown or not applicable cognitive development data, efforts to collect this information are ongoing and may be available as the DMP progresses.

Precision could be interesting as cognition is very frequent in this disease, impacting compliance to some assessments, and as ERT is not known to improve this problem in MPS patients.

- Due to the great heterogeneity of MPSVII patients and the relatively small number recruted here (N=35),  I find more interesting  to add a table with each patient's relevant  data at baseline +/- follow-up when available, or at least with signs/results of assessments (number, pourcentage),  than a table with ethnicity and race.

***Due to the small number of MPS VII patients worldwide (estimated to be 200 to 300 total) and the small number of participants (N=35) currently enrolled in the DMP, including patient-level data may compromise the confidentiality of DMP participants.***

- Could classification of the patients give more accurate interpretable results : for example classification between patients treated before or after puberty ? particularly height evolution data without puberty status has poor value.

***A breakdown of age < 12 years and ≥ 12 years was added to Table 2.***

- Concerning the patient who died due to an accident : was it a motor vehicle accident ? or surgical accident  ? if a short precision is possible.

***This was unfortunately a recreational activity accident and “recreational activity” was added to the text at all mentions of this fatal event.***

- Were patient who experienced  TCSH excluded ? Or no such patient in this  monitoring program ?
- no genetic data ?

***Would the reviewer please kindly provide what is meant by “TCSH”? Genetic mutation data vary across multiple pathogenic variants in these participants and available data were added to Table 2.***

Probable topo error in the name of author 9 : Brigitte Chabrol

***Change made.***

Reviewer #2: The intention of the manuscript to report on RWD is important for the field of lysosomal diseases. The open communication about the inadequate data situation must also be appreciated. Nevertheless, it is difficult to understand what neurological and cognitive findings are present in the patients. A table with patient characteristics could be helpful. The data on treatment with vestronidase are easy to understand and helpful. With regard to lung function, FVC should be emphasised as a restrictive parameter; an obstructive disorder should be represented by the **FVC/FEV1 ratio.** In addition, the extent to which improvements can be explained by learning effects should be commented on. 
The manuscript would also benefit if molecular and genetic data could be compared with a phenotype classification.

***The Pulmonary Function section (page 17) describes the limited availability of pulmonary function data for these participants at this time:***

While the limited data available as of the data cutoff for this report prevents detailed interpretation, the eight participants with post-DMP enrollment visits remain within normal predicted ranges, with stable to slightly improved trends on the volume of air that can be forcibly exhaled in the first second after full inspiration (FEV1) and forced vital capacity (FVC).

***After describing limited preliminary pulmonary data, it is noted:***

The trends observed in FEV1, FVC, and CPF will continue to be closely monitored.

***As more data become available, FVC/FEV1 ratios will be described in a future publication.***

***Genetic mutation data vary across multiple pathogenic variants in these participants and selected data were added to Table 2.***

If improvements to the English language within your manuscript have been requested, you should have your manuscript reviewed by someone who is fluent in English. If you would like professional help in revising this manuscript, you can use any reputable English language editing service. We can recommend our affiliates Nature Research Editing Service ([http://bit.ly/NRES-HS](https://protect-usb.mimecast.com/s/Ei1ICzqgg6fMrXAgTVzRI-?domain=bit.ly)) and American Journal Experts ([http://bit.ly/AJE-HS](https://protect-usb.mimecast.com/s/onaCCvm66QI7lJ3NIo2Jgh?domain=bit.ly)) for help with English usage. Please note that use of an editing service is neither a requirement nor a guarantee of publication. Free assistance is available from our English language tutorial (%CUSTOM_SPRINGER_ENG_LANG_TUTORIAL_URL%) and our Writing resources (%CUSTOM_BMC_WRITING_RESOURCES_URL%). These cover common mistakes that occur when writing in English.


This letter contains confidential information, is for your own use, and should not be forwarded to third parties.

Recipients of this email are registered users within the Editorial Manager database for this journal. We will keep your information on file to use in the process of submitting, evaluating and publishing a manuscript. For more information on how we use your personal details please see our privacy policy at [https://www.springernature.com/production-privacy-policy](https://protect-usb.mimecast.com/s/Ei9_Cwn660tGN5W8I8gxfA?domain=springernature.com). If you no longer wish to receive messages from this journal or you have questions regarding database management, please contact the Publication Office at the link below.

__________________________________________________
In compliance with data protection regulations, you may request that we remove your personal registration details at any time.  (Use the following URL: [https://www.editorialmanager.com/ojrd/login.asp?a=r](https://protect-usb.mimecast.com/s/9smzCxoWW6S1Xp3MT2ogOI?domain=editorialmanager.com)). Please contact the publication office if you have any questions.
